# Supplementary material for: Hybrid Models and Biological Model Reduction with PyDSTool
Source: PLoS Comput Biol. 2012 Aug 9;8(8):e1002628. doi: 10.1371/journal.pcbi.1002628 (PMC3415397; doi:10.1371/journal.pcbi.1002628)
Supplement: Text S4 — Complete source code for the PyDSTool package (version 0.88.120504). Includes API documentation and help files linking to web pages. This file is identical to the current public release on Sourceforge.net. (ZIP) [file pcbi.1002628.s004.zip › PyDSTool/html/PyDSTool.Toolbox.optimizers.line_search.fixed_last_step_modifier.FixedLastStepModifier-class.html]

xml version="1.0" encoding="ascii"?


PyDSTool.Toolbox.optimizers.line\_search.fixed\_last\_step\_modifier.FixedLastStepModifier


| Home | Trees | Indices | Help | PyDSTool |
| --- | --- | --- | --- | --- |

Package PyDSTool ::
Package Toolbox ::
Package optimizers ::
Package line\_search ::
Module fixed\_last\_step\_modifier ::
Class FixedLastStepModifier

[hide private]

[frames] | no frames]

# Class FixedLastStepModifier

source code

```
object --+
         |
        FixedLastStepModifier
```

---

Overrides the default step size and replaces it with a factor times
the last one


|  |  |
| --- | --- |
| Instance Methods | [hide private] |

|  |  |
| --- | --- |
| \_\_init\_\_(self, line\_search, alpha\_factor=2.0, \*\*kwargs)  Needs to have : | source code |

|  |  |
| --- | --- |
| \_\_call\_\_(self, origin, function, state, \*\*kwargs)  Returns a good candidate Parameters : | source code |

**Inherited from `object`**:
`__delattr__`,
`__getattribute__`,
`__hash__`,
`__new__`,
`__reduce__`,
`__reduce_ex__`,
`__repr__`,
`__setattr__`,
`__str__`


|  |  |
| --- | --- |
| Properties | [hide private] |

**Inherited from `object`**:
`__class__`


|  |  |
| --- | --- |
| Method Details | [hide private] |

|  |  |
| --- | --- |
| \_\_init\_\_(self, line\_search, alpha\_factor=2.0, \*\*kwargs)  *(Constructor)* | source code |

Needs to have :

- the decorated line search (line\_search)

Can have :

- an alpha modifier, a factor to modulate the last step length
  (alpha\_factor = 2.)

Overrides: object.\_\_init\_\_

|  |  |
| --- | --- |
| \_\_call\_\_(self, origin, function, state, \*\*kwargs)  *(Call operator)* | source code |

Returns a good candidate Parameters :

- origin is the origin of the search
- function is the function to minimize
- state is the state of the optimizer

  


| Home | Trees | Indices | Help | PyDSTool |
| --- | --- | --- | --- | --- |

|  |  |
| --- | --- |
| Generated by Epydoc 3.0.1 on Fri May 4 15:24:09 2012 | http://epydoc.sourceforge.net |
